# Identification of Mineralocorticoid Receptors, Aldosterone, and Its Processing Enzyme CYP11B2 on Parasympathetic and Sympathetic Neurons in Rat Intracardiac Ganglia

**DOI:** 10.3389/fnana.2021.802359

**Published:** 2022-01-11

**Authors:** Lukas Dehe, Shaaban A. Mousa, Noureddin Aboryag, Mohammed Shaqura, Antje Beyer, Michael Schäfer, Sascha Treskatsch

**Affiliations:** ^1^Department of Anesthesiology and Intensive Care Medicine, Charité Campus Benjamin Franklin, Charité—Universitätsmedizin Berlin, Corporate Member of Freie Universität and Humboldt Universität zu Berlin, Berlin, Germany; ^2^Department of Anaesthesiology, Ludwig-Maximilians-University Munich, Munich, Germany

**Keywords:** heart, aldosterone, receptor, parasympathetic, sympathetic, immunohistochemistry

## Abstract

Recent interest has focused on the mineralocorticoid receptor (MR) and its impact on the myocardium and the performance of the heart. However, there is a lack of evidence about MR expression and its endogenous ligand aldosterone synthesis with specific regard to the intrinsic cardiac nervous system. Therefore, we looked for evidence of MR and aldosterone in sympathetic and parasympathetic neurons of intracardiac ganglia. Tissue samples from rat heart atria were subjected to conventional reverse-transcriptase polymerase chain reaction (PCR), Western blot, and double immunofluorescence confocal analysis of MR, corticosterone-inactivating enzyme 11β-hydroxysteroid-dehydrogenase-2 (11β-HSD2), aldosterone, and its processing enzyme CYP11B2 together with the neuronal markers vesicular acetylcholine transporter (VAChT) and tyrosine hydroxylase (TH). Our results demonstrated MR, 11β-HSD2, and CYP11B2 specific mRNA and protein bands in rat heart atria. Double immunofluorescence labeling revealed coexpression of MR immunoreactivity with VAChT in large diameter parasympathetic principal neurons. In addition, MR immunoreactivity was identified in TH-immunoreactive small intensely fluorescent (SIF) cells and in nearby VAChT- and TH-immunoreactive nerve terminals. Interestingly, the aldosterone and its synthesizing enzyme CYP11B2 and 11β-HSD2 colocalized in MR– immunoreactive neurons of intracardiac ganglia. Overall, this study provides first evidence for the existence of not only local expression of MR, but also of 11β-HSD2 and aldosterone with its processing enzyme CYP11B2 in the neurons of the cardiac autonomic nervous system, suggesting a possible modulatory role of the mineralocorticoid system on the endogenous neuronal activity on heart performance.

## Introduction

Corticosteroids are steroid hormones produced in the adrenal cortex and bind to two types of receptors, the glucocorticoid and mineralocorticoid receptor (Gray et al., 2017). It is well known that the major physiological adrenocorticosteroids, glucocorticoids, and mineralocorticoids including aldosterone, play a crucial role in controlling important cardiovascular functions (Gray et al., 2017). Indeed, the activation of both glucocorticoid receptor (GR) and mineralocorticoid receptor (MR) in the heart affects cardiac development, physiology, and pathophysiology (Oakley and Cidlowski, 2015; Richardson et al., 2016), and this is implicated in the development of cardiovascular remodeling during cardiac fibrosis and heart failure (HF) (Brilla and Weber, 1992; Young and Funder, 1996; Weber, 2001).

Aldosterone, through the activation of MR, apparently is able to modulate parasympathetic effects as previous studies have shown that aldosterone diminished the baroreceptor discharge in dogs (Wang, 1994) and the bradycardic response to pressor stimuli in healthy man (Malik, 1996). Also, aldosterone promotes sympathetic activation (MacFadyen et al., 1997) and augments the sympathetic activity of catecholamines by attenuating their uptake in myocardial tissues (Struthers, 2002). Consequently, MR antagonists not only improve cardiac autonomic function but also reduce left ventricular remodeling and heart rate (Yee and Struthers, 1998; Kasama et al., 2003; Davies et al., 2005).

Aldosterone was classically thought to be synthesized solely in the adrenal cortex. However, recent emerging evidence has demonstrated local synthesis of aldosterone also outside the adrenal gland such as brain (Gomez-Sanchez et al., 1997), peripheral sensory neurons (Mohamed et al., 2020), heart (Silvestre et al., 1998; Funder J. W., 2001), and vessel walls (Takeda et al., 1995, 1997). Consistently, aldosterone synthase expression has also been proposed in the rat (Silvestre et al., 1998; Casal et al., 2003) and human (Young et al., 2001; Tsybouleva et al., 2004) heart. Moreover, in support of the functional importance of such expression, a net release of aldosterone was observed across the human coronary vascular bed (Nakamura, 2004).

On the other hand, it is well accepted that the neuronal control of the heart is under the influence of its own “little brain,” from the parasympathetic and the sympathetic divisions of the autonomic nervous system including the intrinsic cardiac ganglia within the atria (Mousa et al., 2010; Durães Campos et al., 2018). Indeed, a previous study reported a modulatory role of intracardiac ganglia on heart rate (chronotropy), atrioventricular conduction (dromotropy), and myocardial contraction (inotropy) (Adams and Cuevas, 2004). Recently, increasing evidence indicated that MR are known to play physiological and pathophysiological roles in the cardiovascular system, and MR activation directly damages these organs (Funder J., 2001). Direct effects of aldosterone on the heart indeed require the presence of its specific receptor, the MR, in the myocardium. Several experimental studies show that this is the case. Consistently, specific binding of aldosterone to MR (or high-affinity type I sites) has been reported in rat heart. Immunodetection of MR was evidenced in the four cavities of rabbit and human heart by use of H10E, a specific antiidiotypic antibody against MR (Bonvalet et al., 1995; Yoshida et al., 2005).

However, until now, there is no conclusive evidence of the expression and anatomical localization of the mineralocorticoid receptors, its endogenous ligand aldosterone, the enzyme aldosterone synthase CYP11β2, and the MR protecting enzyme 11β-HSD2 with specific regard to the parasympathetic and sympathetic innervations of the heart. Therefore, due to the recent increasing interest of the modulating effects of local mineralocorticoid receptors on the autonomic cardiac nervous system, we set out to systematically examine the expression of MR in rat heart atria containing intracardiac ganglia using conventional PCR and Western blot. Moreover, we aimed at the systematic investigation of the colocalization of MR, 11β-HSD2, and CYP11β2 with specific markers for parasympathetic neurons (vesicular acetylcholine transporter—VAChT) and catecholaminergic (including sympathetic) neurons (tyrosine hydroxylase (TH) by double immunofluorescence confocal microscopy. The results of these findings may provide anatomical evidence of the local mineralocorticoid receptor and its corresponding endogenous ligand for the autonomic regulation of cardiac function.

## Materials and Methods

### Animals

Experiments were conducted in adult male Wistar rats (breeding facility, Charité-Universitätsmedizin Berlin, Germany) after approval by the local animal care committee. Animal care and experiments were performed in accordance with the European Directive introducing new animal welfare and care guidelines (2010/63/EU).

### Tissue Preparation

Rats were deeply anesthetized with isoflurane, and their heart tissue,- including the right and left atria, the left precaval vein, short lengths of the pulmonary veins and the superior and inferior vena cava, were removed from adult rats for conventional PCR (*n* = 5), Western blot (*n* = 5), and immunohistochemistry (*n* = 5).

### Glucocorticoid Receptor, Mineralocorticoid Receptor, 11β-HSD2, and CYP11B2 mRNA Detection by Conventional Polymerase Chain Reaction Analysis

Conventional PCR analysis for GR, MR, 11β-HSD2, and CYP11B2 specific mRNA from rat atria was performed as described previously (Mousa et al., 2010). Tissues of the atria were collected in lysis-buffer. mRNA was extracted using a Qiagen Mini Kit (Qiagen, Hilden, Germany). One microliter of oligo dT was added to 10.4 μl RNA, incubated at 25°C for 10 min, then at 42°C for 60 min, finally at 85°C for 5 min, and then transferred onto ice. cDNA was stored at −80°C. The following specific primers for MR: forward primer, 5′-CCAAGGTAC TTCCAGGATTTAAAAAC-3′, reverse primer, 5′-AACGATGATAGACACATCCAAGAAT ACT-3′ (Ensemble Accession No: NM_013131.1); for aldosterone synthase CYP11B2: forward primer, 5′-TGGCAGC ACTAATAACTCAGGG-3′, reverse primer: 5′-ATTGCTGTCG TGTC AACGCT-3′ (Ensemble, Accession No: NM_012538.2), and for 11β-Hydroxysteroid-Dehydrogenase 2: forward primer, 5′-CGCCGCTTCCTACAGAACTT-3′, reverse primer, 5′-TCCTGGGTTGTGTCATGAACA-3′ (Ensemble Accession No: NM_017081.2). For PCR analysis, Maxima Hotstart Green Enzyme (Thermo Scientific) was used for the subsequent steps. Amplification was carried out for 40 cycles, each consisting of 30 s at 95°C and of 30 s at 60°C and 30 s at 72°C. Specific bands were visualized on an agarose gel by a gel documentation system (EasyDoc, Fa. BioRad).

### Western Blot

Tissues of the right and left atria, including the interatrial septum, were placed in cold buffer and homogenized within 2 h after collection or rapidly frozen in tissue wells placed directly on dry ice and stored at −80°C for subsequent processing. Western blot analysis was performed as previously described (Mousa et al., 2010, 2011). Briefly, the samples were homogenized in boiling SDS sample buffer (100 mM Tris, 2% SDS, 20% glycerol). The protein concentration was measured in a BCA assay (Pierce, Rockford, IL). 2-Mercaptoethanol and bromophenol blue were added before loading. The extracts were separated using SDS-PAGE (10%) with 20 μg protein per lane and then transferred onto nitrocellulose filters. The membranes were blocked in 3% BSA for 2 h and incubated with monoclonal mouse anti-MR (private gift from Celso E. Gomez-Sanchez, University of Mississippi, Jackson, Mississippi), mouse anti-aldosterone synthase (anti-CYP11□2, Merck Millipore, Darmstadt, Germany; 1:1,000 in 3% BSA), or rabbit anti-11β-hydroxysteroid-dehydrogenase 2 (St. John’s Laboratory Ltd., London, United Kingdom; 1:500 in 3% BSA) (Li et al., 2018) overnight at 4°C. After incubation with the secondary antibody (peroxidase-conjugated goat anti-rabbit, 1:40.000, Jackson ImmunoResearch, West Grove, PA) for 2 h at room temperature, reactive protein bands were digitally visualized using ECL solutions (SuperSignal West Pico, Thermo Scientific) by a Fusion Solo S Imager, Fa. Peqlab (Software Vilber Lourmat).

## Immunohistochemistry

### Tissue Preparation

Adult rats were deeply anesthetized with isoflurane and transcardially perfused with 100 ml warm saline, followed by 300 ml of 4% (w/v) paraformaldehyde in 0.16 M phosphate buffer solution (pH 7.4), as previously described (Mousa et al., 2010). After perfusion the atria were removed by cutting along the atrioventricular groove, and the aorta and pulmonary trunk were gently detached. The remaining tissue included the right and left atria, the left precaval vein, short lengths of the pulmonary veins, and superior and inferior vena cava were now also removed and fixed in the same fixative for 90 min, and then cryoprotected overnight at 4°C in PBS containing 10% sucrose. The tissues were then embedded in Tissue-Tek compound (OCT, Miles Inc. Elkhart, IN) and frozen. The tissues were cut tangentially to the atrial wall beginning at the most superior aspect of the atria and ending in the ventricular myocardium at the superior aspect of the right and left bundle branches into 50-μm-thick sections in a cryostat. The sections were collected in PBS (floating sections). In addition, 8-μm-thick sections were mounted on the gelatin-coated slide.

### Double Immunofluorescence Staining

Double immunofluorescence staining was processed as described previously (Mousa et al., 2007). Floating tissue sections or slide-mounted tissue sections were incubated for 60 min in PBS containing 0.3% Triton X-100, 1% BSA, 10% goat serum (Vector Laboratories, CA, United States) (blocking solution) to prevent non-specific binding. Tissue sections were then incubated overnight with the following primary antibodies: (1) polyclonal rabbit anti-MR (private gift from M. Kawata, Koyoto, Japan); this antibody has previously been shown in COS-1 cells with or without MR transfection to be highly specific (Shaqura et al., 2016a) or anti-MR (Santa Cruz), sc-11412; this antibody has previously been proven to be highly specific following MR transfection in different cell lines (Shaqura et al., 2016b) in combination with polyclonal goat anti-VAChT or a monoclonal mouse anti-TH (Immunostar Inc., WI, United States, 1:2,000); (2) polyclonal rabbit antialdosterone (1:500; Novus Biologicals, LLC, United States) in combination with the monoclonal mouse anti-MR (Celso E. Gomez-Sanchez) or aldosterone synthase (anti-CYP11B2,Merck Millipore, Darmstadt, Germany; 1:500); (3) mouse monoclonal antimineralocorticoid receptor (private gift from Dr. Elise Gomez-Sanchez) in combination with polyclonal sheep antibody against 11β-hydroxysteroid-dehydrogenase.

After incubation with primary antibodies, the tissue sections were washed with PBS and then incubated with Alexa-Fluor 594 donkey anti-rabbit antibody (Vector Laboratories) in combination with Alexa Fluor 488 goat anti-guinea pig, anti-mouse, anti-chicken, or anti-sheep antibody (Invitrogen, Germany). Thereafter, sections were washed with PBS, and the nuclei were stained bright blue with 4′-6-diamidino-2-phenylindole (DAPI) (0.1μg/ml in PBS) (Sigma). Finally, the tissues were washed in PBS, mounted in vectashield (Vector Laboratories), and imaged on a confocal laser scanning microscope, LSM510, equipped with an argon laser (458/488/514 nm), a green helium/neon laser (543 nm), and a red helium/neon laser (633 nm; Carl Zeiss, Göttingen, Germany) as described previously (Li et al., 2018). To demonstrate specificity of staining, the following controls were included as described in our previous studies (Li et al., 2018): omission of either the primary antisera or the secondary antibodies. Single optical slice images were taken using × 20 Plan-Neofluar air interface or × 40 Plan-Neofluar oil interface objective lens with a confocal laser scanning microscope, LSM510, equipped with an argon laser (458/488/514 nm), a green helium/neon laser (543 nm), and a red helium/neon laser (633 nm; Carl Zeiss, Göttingen, Germany).

### Quantification of Immunostaining

For the quantitative evaluation of all immunohistochemical stainings, the version 1.41 of the image analysis program ImageJ^®^ was applied^1^ (Shimojo et al., 1997; Schneider et al., 2012). The additional use of the plug-in (color deconvolution) allowed the separation of the different color channels, each identifying distinct target structures, whose color signal can, thus, be quantitatively evaluated. A manually specified area was identified for each specifically colored area. Intensity thresholds were assigned, so that a maximum degree of integrated area of stained target structure was identified, while minimizing possible background activities. Areas above the threshold value were, thus, defined as positive and indicated information about the percentage of the immunostained area in relation to the previously selected total area. Values below the threshold were eliminated as background. The threshold value was kept constant for all sections of staining. With the help of ImageJ, the parameter percentage area (% stained area) was calculated using the software. The percentage area was defined as the specific colored area in relation to the total area of a photographed tissue preparation. All calculated quantitative color intensities are presented as percentage of immunoreactive area in the manuscript (see also Supplementary Figure 1).

## Results

### Identification of Mineralocorticoid Receptor, 11β-HSD2, and CYP11B2 Specific mRNA and Proteins in Rat Atria

**FIGURE 1:**
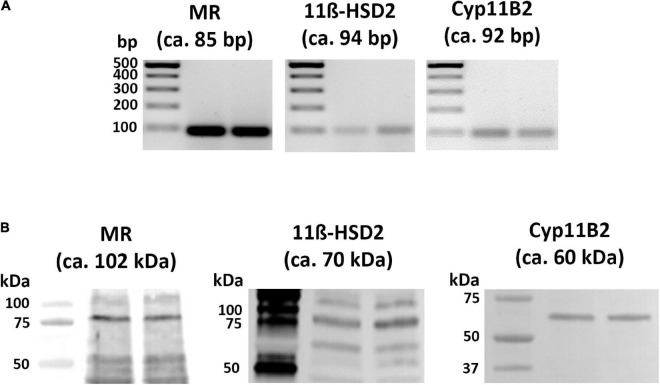
Detection of MR, 11β-HSD2, and CYP11B2 mRNA **(A)** and proteins **(B)** in rat atria by conventional PCR analysis **(A)** and western blot **(B)**, respectively. **(A)** MR, 11β-HSD2, and CYP11B2 mRNA were reverse transcribed into cDNA and amplified by conventional PCR; subsequent gel electrophoresis of PCR products shows the expected 85-bp fragment for MR, 94-bp fragment for 11β-HSD2, and 92-bp fragment for CYP11B2 in rat heart atria. **(B)** Western blot analysis of MR, 11β-HSD2, and CYP11B2 protein bands with mouse monoclonal anti-MR (lanes 1 and 2), rabbit polyclonal anti-11β-HSD2 (lanes 3 and 4), and rabbit polyclonal anti-CYP11B2 (lanes 5 and 6) in rat atria show protein bands with the expected molecular weights of 102 kDa for MR, 70 kDa for 11β-HSD2, and 60 kDa for CYP11B2 in addition to several other molecular weight bands.

Using distinct primer pairs and conventional reverse transcriptase PCR analysis, the expected MR, 11β-HSD2, and CYP-11B2 specific PCR-products (Figure 1A) were identified in the tissue of the right and left atria of naïve rats. Gel electrophoresis of these PCR products showed the expected 85-bp cDNA fragment for MR, 94-bp cDNA fragment for 11β-HSD2, and 92-bp cDNA fragment for CYP11B2 (Figure 1A).

Using specific antibodies against MR, 11β-HSD2, and CYP11B2, gel electrophoresis with subsequent immunoblots of tissue extracts from rat atria consistently showed the predicted MR, 11β-HSD2, as well as CYP11B2 (Figure 1B) specific protein bands at the expected molecular weights of 102, 70, and 60 kDa, respectively in addition to several other molecular weight bands.

### Localization of Mineralocorticoid Receptor in Cardiac Parasympathetic Vesicular Acetylcholine Transporter-Immunoreactive Neurons Within Rat Atria

**FIGURE 2:**
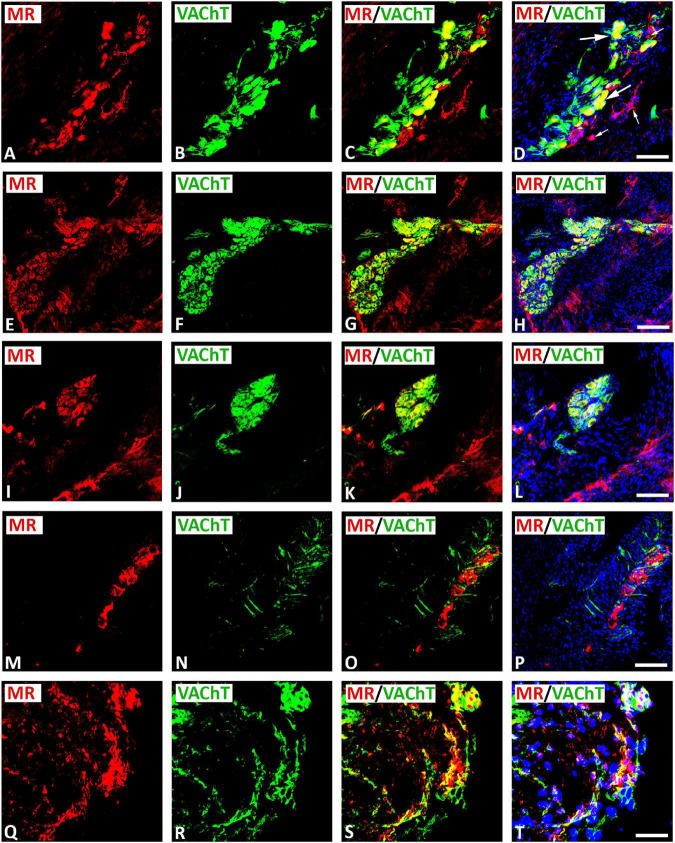
Double immunofluorescence confocal microscopy of typical intracardiac ganglia neurons coexpressing MR **(A,E,I,M,Q)** (*red fluorescence*) and VAChT **(B,F,J,N,R)** (*green fluorescence*). **(A–L)** Show MR colocalizing with the parasympathetic neuronal marker VAChT in large diameter spherical neurons (long arrow); however, some small diameter neurons expressed MR only (small arrow). Note, VAChT immunoreactivity was also observed in pericellular boutons surrounding MR–IR cell bodies of most principal neurons **(E–L)**. **(M–P)** Show that MR was expressed on clusters of spherical and small diameter neurons surrounded with dispersed single fibers exclusively positive for VAChT. **(Q–T)** Show a cluster of intracardiac neurons coexpressing MR and VAChT within gelatin-embedded transverse sections. **(D,H,L,P,T)** Show DAPI nuclear staining (bright blue). Bar = 20 μm.

**FIGURE 3:**
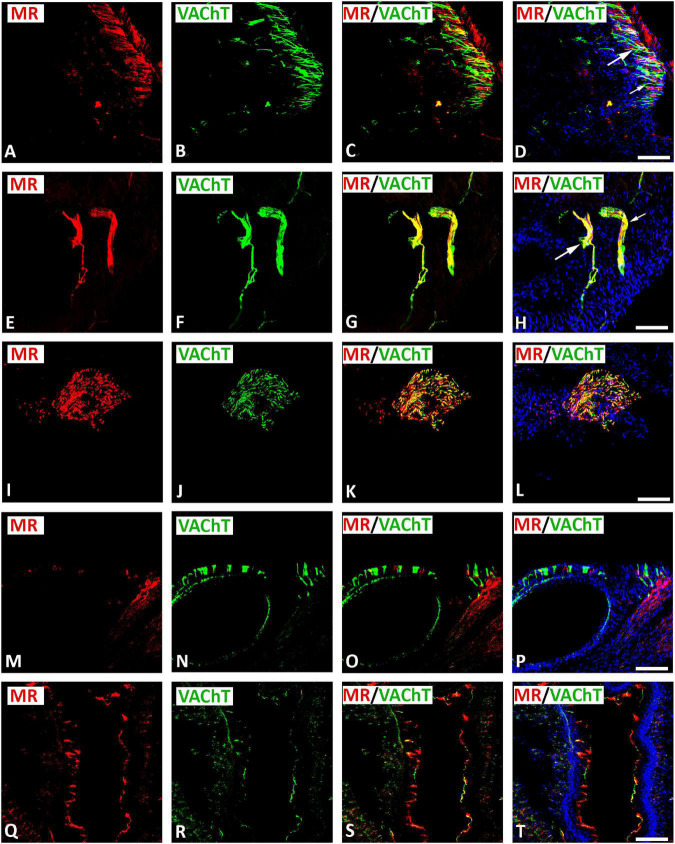
Double immunofluorescence confocal microscopy of nerve fibers expressing MR **(A,E,I,M,Q)** (red fluorescence) and/or VAChT **(B,F,J,N,R)** (green fluorescence) within rat heart atria. **(A–D)** Show MR immunoreactivity co-localizing with VAChT-IR in a network of axons within rat atria. **(E–H)** Show epicardial bundles of non-varicose (small arrow) or varicose (large arrow) VAChT-positive fibers co-expressing MR immunoreactivity. Note, some fiber expressed MR or VAChT only. **(I–L)** Represent crosssectioned epicardiac nerves within heart atria containing a high population of VAChT-IR fibers colocalizing with MR-immunoreactivity. **(M–P)** Thick non-varicose VAChT-positive terminals protruding vertically into the endothelium superior the vena cava with few fibers being positive for MR. **(Q–T)** Show thin MR-positive terminals penetrating the atrial endocardium that are additionally positive for VAChT-immunoreactiviy. **(D,H,L,P)** Show DAPI nuclear staining (bright blue). Bar = 20 μm.

Double immunofluorescence confocal microscopy identified MR immunoreactivity on some subpopulations of VAChT-immunoreactive (IR) large diameter parasympathetic neurons of intracardiac ganglia (Figure 2). Some VAChT-IR neurons lacked MR immunoreactivity and vice versa (Figures 2A–D). VAChT immunoreactivity was also observed in pericellular preganglionic boutons surrounding MR–IR cell bodies of most principal neurons (Figures 2E–L). In tissue sections of rat atria, quantification of the median[range]% values of the area of MR colocalizing with VAChT (yellow fluorescence) revealed up to 34[10–38]% overlap, whereas 64% did not (red fluorescence only). Moreover, 34[13–61]% of immunoreactive area of double staining of VAChT colocalized with MR (yellow fluorescence), whereas 64% did not (green fluorescence only). In addition, a thick cardiac nerve consisting of non-varicose or varicose VAChT-positive nerve fibers coexpressing MR immunoreactivity was identified in rat atria (Figures 3A–H). Some VAChT-IR nerve fibers lacked MR immunoreactivity and vice versa (Figures 3A–D). Consistently, crosssections of epicardiac nerves within heart atria containing high populations of VAChT-IR fibers expressed MR immunoreactivity (Figures 3I–L). Additionally, thick non-varicose VAChT-positive terminals protruding vertically into the endothelium superior vena cava with few fibers were positive with MR immunoreactivity (Figures 3M–P). Also, thin and varicose MR-positive nerve terminals penetrating the endocardial surface of atrium were positive with VAChT (Figures 3Q–T). MR were not only expressed in nerve fibers, but also in the myocardium of atria (Figures 3M–P).

### Localization of Mineralocorticoid Receptor in Sympathetic Tyrosine Hydroxylase–Immunoreactive Neurons Within Rat Atria

**FIGURE 4:**
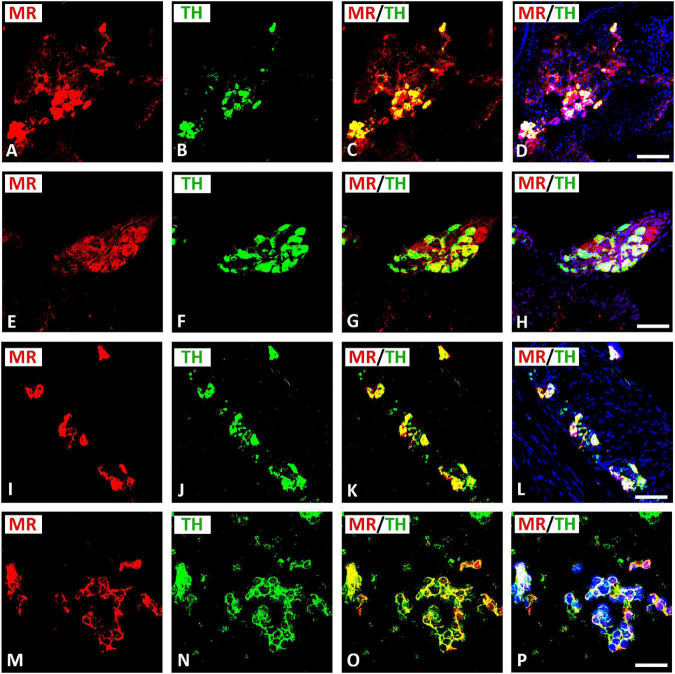
Confocal microscopy of MR **(A,E,I,M)** (red fluorescence) and TH **(B,F,J,N)** (green fluorescence) double immunofluorescence in intracardiac ganglia. **(A–H)** Show MR coexpressing TH in small intensely fluorescent (SIF) cells but not in large-diameter neurons, previously identified by McMahan and Purves (McMahan and Purves, 1976; Heym et al., 1994), however, some SIF cells also expressed TH only. **(I–L)** Show dispersed intracardiac neurons within rat atrium being not positive for VAChT but coexpressing TH and MR in adjacent sections (see Figures 2M–P). **(M–P)** Higher magnification of gelatine mounted tissue sections of atria. Note a high majority of SIF cells coexpressing MR with TH; however, few SIF cells also expressed TH only. **(D,H,L,P)** Show DAPI nuclear staining (bright blue). Bar = 20 μm.

**FIGURE 5:**
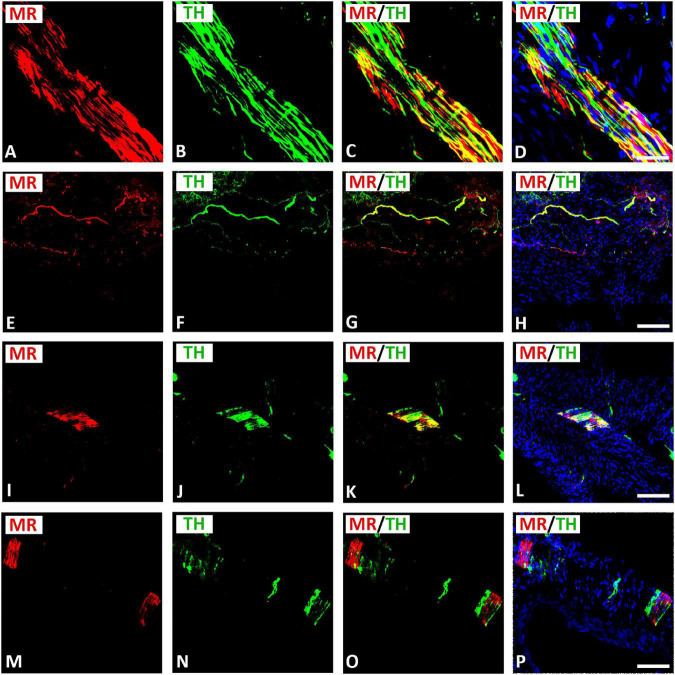
Confocal microscopy of MR **(A,E,I,M,Q)** (red fluorescence) and TH **(B,F,J,N,R)** (green fluorescence) double immunofluorescence of nerve fibers within rat atria. **(A–H)** Show MR expressing TH-IR bundles **(A–D)** or single axons **(E–H)** of epicardial nerves within rat atria. Note, some fibers expressed MR or TH only. **(I–P)** Show bundles of axons of epicardial nerves within rat atria positive for MR and heavily **(I–L)** or rarely **(M–P)** positive for TH. **(D,H,L,P)** Show DAPI nuclear staining (bright blue). Bar = 20 μm.

Double immunofluorescence confocal microscopy identified MR immunoreactivity in TH–IR small (5–10 μm) intensely fluorescent (SIF) cell-like neurons of intracardiac ganglia; however, some SIF cells were positive for TH only (Figure 4). SIF cells could be distinguished from large-diameter principal neurons that were positive for MR, but did not colocalize with TH (see Figures 4A–H). The majority of SIF cells expressing TH immunoreactivity was lacking VAChT (see Figures 2M–P). In gelatin-embedded atrial tissue sections, the anti-MR antibody stained many small cells within intracardiac ganglia that had round or spheroid cell somata with diameters ranging from 10 to 20 μm (Figures 4M–P). In tissue sections of rat atria, quantification of the median[range]% values of the area of MR colocalizing with TH (yellow fluorescence) revealed up to 58[28–79]% overlap, whereas 42% did not (red fluorescence only). Moreover, 15[49–80]% of immunoreactive area of double staining of TH colocalized with MR (yellow fluorescence), whereas 85% did not (green fluorescence only). In addition, MR immunoreactivity was identified in some TH–IR course bundle fibers of epicardial nerves, intensely innervating rat heart atria (Figures 5A–D). Some TH immunoreactive fibers lacked MR immunoreactivity and vice versa. Some epicardial nerves contained exclusively TH–IR fibers next to adjacent MR-positive fibers (Figures 5E–P).

### Coexpression of Mineralocorticoid Receptor With Its Protecting Enzyme 11β-HSD2 as Well as Aldosterone With Its Processing Enzyme CYP11β2 in Cardiac Neurons Within Rat Atria

**FIGURE 6:**
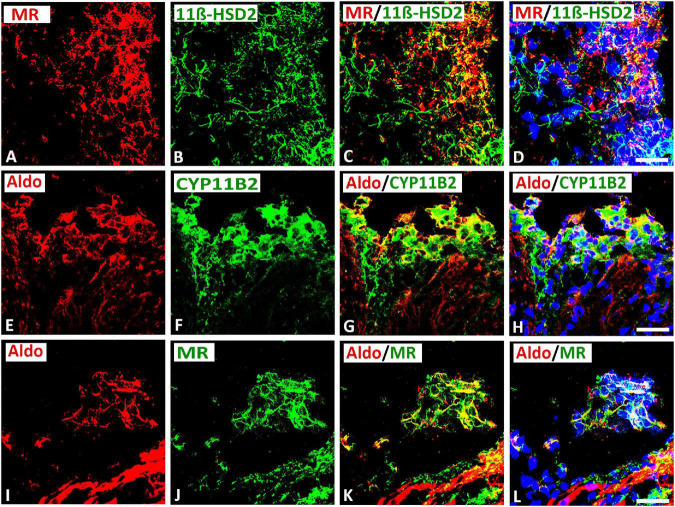
Confocal microscopy of double immunofluorescence of MR **(A)** (red fluorescence) with cortisol-inactivating enzyme 11β-hydroxysteroid-dehydrogenase -2 (11β-HSD2) **(B)** (green fluorescence) in intracardiac ganglia as well as aldosterone **(E,I)** (red fluorescence) with CYP11B2 **(F)** or MR **(J)** (green fluorescence). **(A–D)** Show double immunofluorescence of MR with 11β-HSD2 in intracardiac ganglia. Note that the majority of MR–IR neurons coexpress 11β-HSD2; however, some neurons express MR or 11β-HSD2 alone or vice versa. **(E–L)** Show that aldosterone immunoreactivity colocalized in the majority of CYP11B2- **(F)** or MR- **(J)** immunoreactive (IR) neurons; however, some neuronal cells express aldosterone alone or vice versa. Note a clear localization of aldosterone immunoreactivity with CYP11B2- or MR-IR neuronal cells. Bar = 20 μm.

Double immunofluorescence confocal microscopy of gelatin-embedded atria tissue sections showed that some MR colocalized with its protecting enzyme 11β-HSD2 in up to 30[24–35]% (median[range]%) of immunoreactive area of double staining in the intracardiac neuron (Figures 6A–D). Since aldosterone is derived from a final conversion of 18-hydroxycorticosterone into aldosterone by aldosterone synthase CYP11B2, we examined the local expression of this enzyme in intracardiac neurons. Indeed, double immunofluorescence confocal microscopy of gelatin-embedded atrium tissue sections revealed that aldosterone colocalized with its key processing enzyme CYP11B2 up to 37[35–39]% (median[range]%) of immunoreactive area of double staining in the neuronal population of intracardiac ganglia (Figures 6E–H). Also, some aldosterone immunoreactivity colocalized with MR in up to 30[12–34]% (median[range]%) of immunoreactive area of double staining in the intracardiac ganglia within rat atria (Figures 6I–L). Additionally, MR was expressed in atrial myocardium (data not shown).

## Discussion

Our experiments identified mRNA and proteins specific for MR, its protecting enzyme 11β-HSD2, as well as the aldosterone synthesizing enzyme CYP11B2 in the right and left atria of the rat. Double-immunofluorescence microscopy revealed that MR and its protecting enzyme 11β-HSD2 were densely located on neurons and nerve fibers of the intrinsic cardiac nervous system. More specifically, MR colocalized with parasympathetic VAChT-IR neurons of intracardiac ganglia as well as with sympathetic TH-IR neurons and SIF cell-like neurons of intracardiac ganglia. Intriguingly, the endogenous ligand aldosterone and its synthesizing enzyme CYP11B2 were also detected in these MR–IR neurons. These findings may provide first anatomical evidence for the expression of MR, and also of 11β-HSD2 and the endogenous ligand aldosterone with its processing enzyme CYP11B2 within neurons of the cardiac autonomic nervous system. They may suggest a modulatory role on the endogenous sympathetic and parasympathetic neuronal activity on heart performance.

Using conventional reverse transcriptase PCR, we were able to isolate MR-specific transcripts in rat heart atria. Similar to our previous works in sensory dorsal root ganglia (Shaqura et al., 2016a,b; Li et al., 2018), we identified the expected 85-bp fragment for MR also in rat heart atria. These findings are in line with earlier reports which detected MR transcripts in the rat heart by Northern blot analysis (Arriza et al., 1987; Reul et al., 1989). Consistently, binding sites specific for radiolabeled [3H]-aldosterone were detected in the heart (Funder J. W., 2003). Our western blot analyses recognized the predicted protein bands in rat atria in addition to several smaller bands. Similar results were obtained previously showing multiple additional bands that apparently represent degradation products (Gomez-Sanchez et al., 2011) or posttranslational modifications, particularly glycosylations, which may account for these size variations (Alnemri et al., 1991).

To investigate the exact anatomical localization of MR within rat atria, we performed double immunofluorescence confocal microscopy using specific markers for parasympathetic (VAChT) and sympathetic (catecholaminergic) (TH) neurons. Indeed, our double immunofluorescence analysis demonstrated a colocalization of MR immunoreactivity with VAChT in large diameter parasympathetic principal neurons and VAChT-IR varicose nerve fibers within intracardiac ganglia of the rat heart atria. Regarding the sympathetic innervations of the rat heart, MR also coexpressed with TH in SIF TH–IR paraneuronal cells and sympathetic nerve fibers that course through rat heart atria. These TH–IR neurons are either postsynaptic sympathetic neurons or catecholaminergic paraneuronal cells within intracardiac ganglia, which modify the synaptic transmission of parasympathetic neurons (Adams and Cuevas, 2004). Indeed, previous studies (Struthers, 2004) showed that chronic activation of MR by aldosterone inhibited the baroreceptor heart rate response to infused noradrenaline and, thus, increased sympathetic neuronal activity. In addition to parasympathetic and catecholaminergic neurons, MR was also expressed in atrial myocardium. Our confocal microscopy analysis also confirmed previous studies (Lombès et al., 1992, 1995), which showed that MR was localized in cardiomyocytes, endothelial cells, and large vessels. Its overexpression in a mouse model of pressure-overload-induced heart failure increased MR target gene and protein expression in the heart (Ayuzawa et al., 2016). The present work, showing the localization of MR on neurons of the intracardiac ganglia, provides the starting point for a novel understanding of the control of the intrinsic neuronal system of the heart through corticosteroids. Indeed, Kasama et al. (2003, 2011, 2013) revealed that MR antagonist spironolactone not only enhances left ventricular remodeling, but also increases cardiac sympathetic nerve activity in patients with dilated cardiomyopathy. Also, aldosterone promotes sympathetic activation, and parasympathetic inhibition (Thomas and Marks, 1978; Wang, 1994; MacFadyen et al., 1997). Moreover, aldosterone appears to exert parasympathetic effects as evidenced by the reduction of baroreceptor discharge in the dog (Wang, 1994) and the bradycardic response to pressor stimuli in healthy man (Malik, 1996). The acute perfusion of isolated carotid sinus with aldosterone reduced baroreceptor activity through local MR activation (Wang et al., 1992). Similarly, chronic or systemic application of aldosterone also depressed baroreflex function (Wang, 1994).

It is well accepted that 11β-HSD2 is expressed alongside the MR within mineralocorticoid target tissues, where its activity reduces the access of glucocorticoids, facilitating aldosterone to compete for binding to the MR (Edwards et al., 1988; Funder et al., 1988). In cardiomyocytes, it has been suggested that 11β-HSD2 is expressed at low levels, and MR occupancy by glucocorticoids may predominate (Rusvai and Náray-Fejes-Tóth, 1993; Farman and Rafestin-Oblin, 2001; Viengchareun et al., 2007). In this context, the present work identified 11β-HSD2 specific mRNA and protein in rat atria. In line with previous reports using CHO cells (Náray-Fejes-Tóth and Fejes-Tóth, 1996), western blot analyses recognized the predicted protein band at approximately 70 kDa in rat atria in addition to several other molecular weight bands that may be due to differential posttranslational modifications such as glycosylation (Náray-Fejes-Tóth and Fejes-Tóth, 1996). Indeed, the *in vivo* 11β-HSD2 enzyme activity may be regulated through the formation of inactive dimers in rat kidney (Gomez-Sanchez and Gomez-Sanchez, 2001; Gomez-Sanchez et al., 2003). Alternatively, Shimojo et al. (1997) suggested that the second band in human kidney reflects a modified nuclear 11β-HSD2 protein. Moreover, our results confirmed a colocalization of 11β-HSD2 with MR immunoreactivity in intracardiac ganglion neurons within rata atria. These findings are in line with previous reports by Bonvalet et al. (1995) who detected 11β-hydroxysteroid dehydrogenase activity in the human heart. Recent accumulating evidence argues that the synthesis of aldosterone outside the adrenal gland may contribute to local paracrine effects within the heart (Silvestre et al., 1998; Funder J. W., 2001; Mizuno et al., 2001) and brain (Colombo et al., 2006; Oshima et al., 2013). However, in the rat heart, there is little evidence that aldosterone synthase (CYP11B2) is expressed, and this finding is controversial (Ye et al., 2005; Schiffrin, 2006). In the present work, both CYP11B2 mRNA transcripts and specific protein bands together with the immunohistochemical demonstration of aldosterone in rat atria seem to support the notion that the endogenously synthesized aldosterone within the heart may act in a putative paracrine way on heart performance (Lijnen and Petrov, 2000). This is partially in line with previous studies (Ohtani et al., 2007) which detected cardiac aldosterone by the use of a liquid chromatographic–mass spectrometric method concomitant with increased ventricular levels of MR in diastolic heart failure; however, aldosterone synthase activity and CYP11B2 mRNA were undetectable. The present work, therefore, provides evidence of local aldosterone synthesis in intracardiac neurons within rat heart atria. Aldosterone is known to augment the sympathetic activity of catecholamines leading to cardiac autonomic dysfunction (Struthers, 2002). It also reduces parasympathetic action *via* stimulation of Na + /K + /ATPase and consequently attenuates baroreceptor activity (Davies et al., 2005). It is for these reasons that the MR antagonists improve cardiac autonomic function, reduce heart rate, and attenuate left ventricular remodeling (Yee and Struthers, 1998; Kasama et al., 2003). Taken together, our findings suggest an important role of endogenous aldosterone in the modulation of the autonomic cardiac nervous system.

In summary, immunofluorescence confocal microscopy allowed for the precise anatomical localization of MR, 11β-HSD2, and aldosterone with its processing enzyme CYP11B2 predominantly within the intracardiac ganglia of rat atria. Consequently, we were able to identify MR in VAChT-IR large diameter principal neurons and small TH-IR SIF cells within intracardiac ganglia and atrial myocardium. Interestingly, immunofluorescence confocal microscopy showed coexistence of endogenous ligand aldosterone with its key processing enzyme CYP11B2 and MR located on 11β-HSD2-IR neurons. Our findings can be seen as a step toward a better understanding of the possible role of MR and its endogenous ligand aldosterone on the intrinsic cardiac autonomous nervous system, which might imply a functional role under cardiac disease states.

## Data Availability Statement

The raw data supporting the conclusions of this article will be made available by the authors, without undue reservation.

## Ethics Statement

The animal study was reviewed and approved by the Landesamt für Gesundheit und Soziales, LaGeSo Berlin.

## Author Contributions

SM, LD, ST, and MiS designed the experiments. SM, LD, NA, and MoS performed the experiments. SM, LD, NA, AB, MiS, and ST performed the analyses and interpretation of the experiments. SM, LD, MoS, AB, and ST wrote part of the manuscript. All authors critically reviewed the manuscript.

## Conflict of Interest

The authors declare that the research was conducted in the absence of any commercial or financial relationships that could be construed as a potential conflict of interest.

## Publisher’s Note

All claims expressed in this article are solely those of the authors and do not necessarily represent those of their affiliated organizations, or those of the publisher, the editors and the reviewers. Any product that may be evaluated in this article, or claim that may be made by its manufacturer, is not guaranteed or endorsed by the publisher.

## Footnotes

1
http://rsbweb.nih.gov/ij/

## Supplementary Material

Supplementary Figure 1 Example of quantitative evaluation of immunohistochemical stainings within rat atria using the version 1.41 of the image analysis program ImageJ^®^ (http://rsbweb.nih.gov/ij/). The additional use of the plug-in (color deconvolution) allowed the separation of the different color channels each identifying distinct target structures, whose color signal can, thus, be quantitatively evaluated. With the help of ImageJ, the parameter percentage area (% stained area) was calculated. The percentage area was defined as the specific-colored area in relation to the total area of a photographed tissue preparation.
