# Supplementary figures and images for: Identification of Mineralocorticoid Receptors, Aldosterone, and Its Processing Enzyme CYP11B2 on Parasympathetic and Sympathetic Neurons in Rat Intracardiac Ganglia
Source: Front Neuroanat. 2022 Jan 11;15:802359. doi: 10.3389/fnana.2021.802359 (PMC8786913; doi:10.3389/fnana.2021.802359)

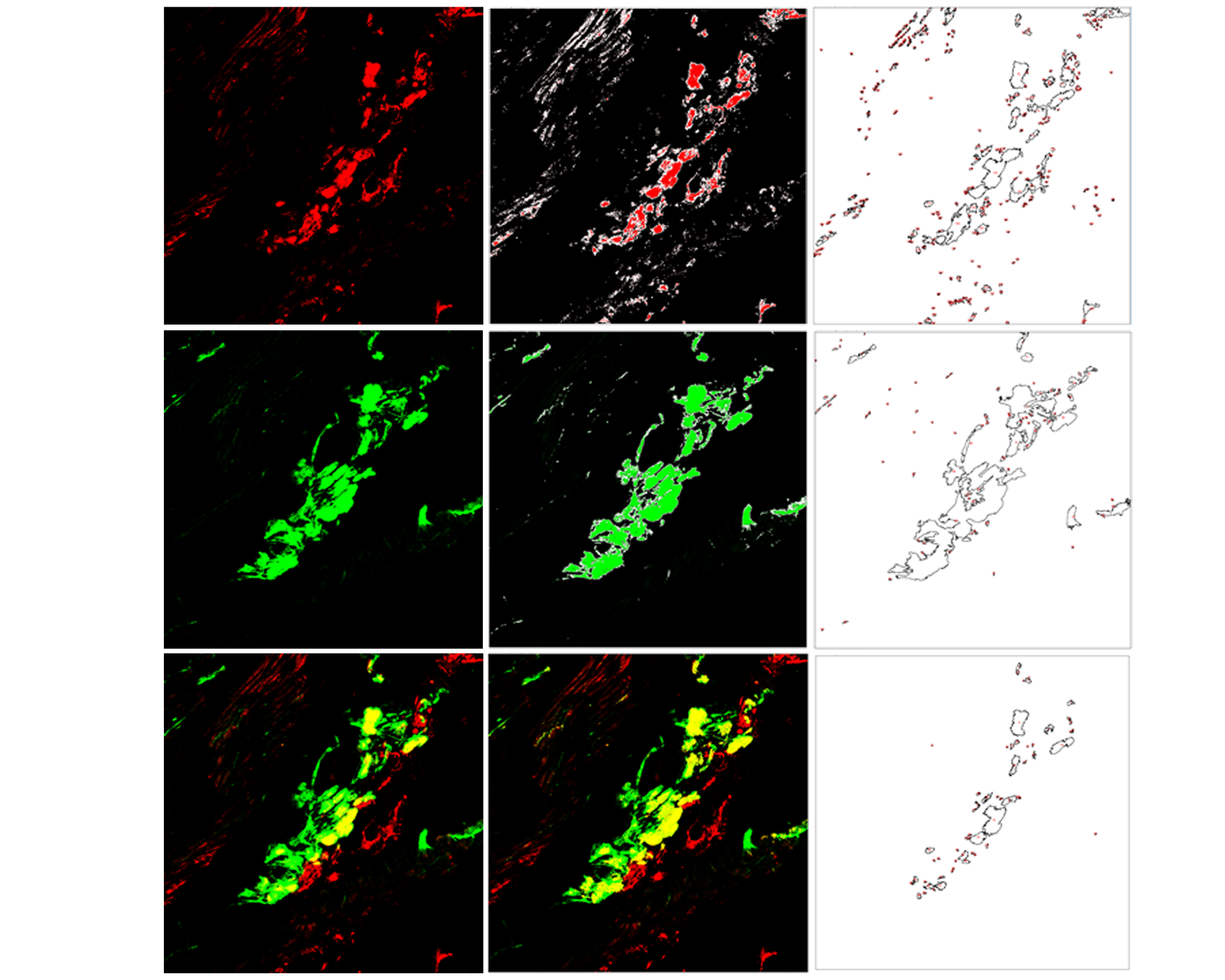

Supplement: Supplementary Figure 1 — Example of quantitative evaluation of immunohistochemical stainings within rat atria using the version 1.41 of the image analysis program ImageJ® (http://rsbweb.nih.gov/ij/). The additional use of the plug-in (color deconvolution) allowed the separation of the different color channels each identifying distinct target structures, whose color signal can, thus, be quantitatively evaluated. With the help of ImageJ, the parameter percentage area (% stained area) was calculated. The percentage area was defined as the specific-colored area in relation to the total area of a photographed tissue preparation. [file Image_1.TIF]
